# Fermented protein foods as modulators of the gut-muscle axis: mechanisms, evidence, and future directions

**DOI:** 10.1038/s42003-026-10941-2

**Published:** 2026-09-25

**Authors:** Dominic N. Farsi, Paul D. Cotter, Orla O’Sullivan

**Affiliations:** 1 Food Biosciences, Teagasc Food Research Centre, Fermoy, Co. Cork Ireland; 2 VistaMilk, Fermoy, Co. Cork Ireland; 3 Department of Nutritional Sciences, King’s College London, London, UK; 4 Centre for Public Health, Queen’s University Belfast, Belfast, UK; 5 APC Microbiome Ireland, University College Cork, Cork, Co. Cork Ireland; 6 School of Cell Biology and Biochemistry, University College Cork, Cork, Co. Cork Ireland

**Keywords:** Skeletal muscle, Applied microbiology, Microbiota

## Abstract

Skeletal muscle has a profound influence on metabolic health, functional capacity and resilience across the lifespan. Beyond dietary protein and physical activity, the gut microbiome may have the capacity to impact skeletal muscle mass and function through the bidirectional network termed the gut-muscle axis (GMA). Fermented protein foods (FPFs) are protein rich matrices transformed by microbial activity that integrate modified protein structures, bioactive peptides, and live microorganisms, or components thereof, which could modulate skeletal muscle physiology. Here, we review mechanistic, preclinical and human evidence concerning the potential of FPFs to influence skeletal muscle health through GMA modulation. Although emerging human studies suggest favourable effects on metabolic regulation, inflammatory pathways and gut microbial ecology, direct evidence that FPFs impact muscle protein synthesis, muscle mass or function remains limited. Furthermore, the literature is characterised by considerable heterogeneity in interventions and outcome measures. Studies integrating comprehensive microbiome characterisation, multi-omics approaches and direct skeletal muscle phenotyping are needed to determine whether FPFs confer advantages over conventional protein foods or more established microbiome targeted strategies such as probiotic supplementation. Such studies will be essential to elucidate their role in targeted nutritional strategies for ageing, metabolic disease, muscle atrophy, and physical performance.

## Introduction

Skeletal muscle is central to metabolic health, functional capacity, and quality of life across the lifespan. Beyond its role in movement and physical performance, skeletal muscle serves as a primary site of glucose disposal, amino acid storage, and systemic metabolic regulation^1,2^. Age-related declines in skeletal muscle mass and function, which can culminate in sarcopenia, a progressive disorder characterised by low muscle mass, strength, and functional performance^3,4^, are associated with frailty, metabolic dysfunction, loss of independence, as well as cognitive decline and increased mortality^1,5–7^. Conversely, in athletic and physically active populations, optimisation of muscle protein turnover can aid in adaptation, recovery, and performance^8^. Thus, strategies that preserve or enhance skeletal muscle mass and function have broad clinical, public health, and performance implications.

Nutritional stimuli, particularly dietary protein intake and delivery of amino acids, remain foundational for supporting muscle protein synthesis (MPS) and mitigating anabolic resistance in ageing^9,10^. However, emerging evidence suggests that muscle metabolism is not regulated solely by nutrient availability and mechanical loading. Increasing attention has focused on the gut-muscle axis (GMA), pertaining to the bidirectional interactions between the gut microbiome and skeletal muscle physiology^11,12^. Preclinical, observational, and early human studies indicate that gut microbial composition and function may influence skeletal muscle mass, strength, mitochondrial function, and inflammatory tone through production of metabolites such as short-chain fatty acids (SCFAs), modulation of immune signalling, and interactions with host anabolic pathways^12,13^. For example, a recent study identified *Roseburia Inulinivorans* as positively associated with muscle strength in humans, which was reduced in abundance in older adults, while supplementation in mice enhanced forelimb grip strength, coinciding with changes in signalling pathways pertinent to skeletal muscle physiology, increases in muscle fibre size and conversion from type I to type II fibres^14^. Although an evolving field, this paradigm disrupts the determinants of skeletal muscle health beyond classical nutrition and exercise stimuli, to include a microbial influence.

It is well understood that diet is a primary modulator of the gut microbiome, and among dietary components, fermented protein foods (FPFs) represent a unique category. Fermented foods (FFs) were defined in 2021 as “foods made through desired microbial growth and enzymatic conversions of food components”^15^. This microbial metabolism generates organic acids, alcohols, gases, and a wide array of secondary metabolites, while often retaining viable microorganisms within the final product^16^. Fermentation not only enhances preservation and sensory attributes but also modifies the structural and biochemical properties of the food matrix. As introduced here, FPFs refer to FFs in which protein constitutes a substantial proportion of total energy or macronutrient content and that meaningfully contribute essential amino acids to the diet. These include fermented dairy products (e.g., yoghurt, kefir, cheese), fermented legumes and soy-based foods (e.g., tempeh), and emerging plant-based protein alternatives derived from substrates such as pea, fava bean, wheat or oat protein. When subjected to fermentation, these protein-rich matrices combine intact or partially hydrolysed proteins with live microbes, microbial components and fermentation-derived bioactive compounds. Specifically, regarding protein, the proteolytic systems of fermentative microorganisms, particularly lactic acid bacteria (LAB), cleave native proteins into smaller peptides and free amino acids, which can potentially enhance digestibility, alter amino acid availability, and generate bioactive peptides with potential physiological activity^16–19^. Importantly, fermentation does not merely add microorganisms to an existing protein source; rather, it can fundamentally remodel the protein matrix, simultaneously altering protein digestibility, amino acid release kinetics, peptide composition, microbial ecology and metabolite profiles. Consequently, FPFs should be considered biologically distinct from their non-fermented counterparts, with the potential to influence skeletal muscle physiology through integrated nutritional and microbiome-mediated mechanisms rather than protein provision alone.

Despite growing interest in the GMA and substantial research on fermented dairy and cardiometabolic health, no review to date has directly focused on the evidence linking FPFs to skeletal muscle physiology via the GMA. Existing evidence remains fragmented across studies of FFs, probiotic (defined as “live microorganisms that, when administered in adequate amounts, confer a health benefit on the host”^20^) supplementation trials, and mechanistic investigations of gut-derived metabolites, although the microbial composition and physiological effects of FFs may differ substantially from isolated probiotic interventions. Unlike probiotic supplements, which typically deliver one or a limited number of microbial strains, FPFs provide a complex biological matrix comprising modified proteins, fermentation-derived peptides, microbial metabolites and, in some cases, viable microorganisms. The physiological effects of FPFs are therefore likely to arise from interactions among these components, rather than from microbial delivery alone, providing a mechanistic rationale for considering FPFs as a distinct nutritional strategy for modulating the GMA.

Accordingly, the present review aims to integrate these domains by (1) outlining mechanistic pathways through which FPFs may influence skeletal muscle, (2) evaluating the current landscape of human evidence investigating the effects of FPFs on skeletal muscle-related outcomes, and (3) identifying current knowledge gaps and future research priorities. By framing FPFs within the context of the GMA, we propose a conceptual framework for understanding how fermentation-induced changes to protein foods may interact with host-microbe crosstalk to influence skeletal muscle physiology and to guide future mechanistic and clinical research.

## Mechanistic basis of the gut-muscle axis

**Fig. 1:**
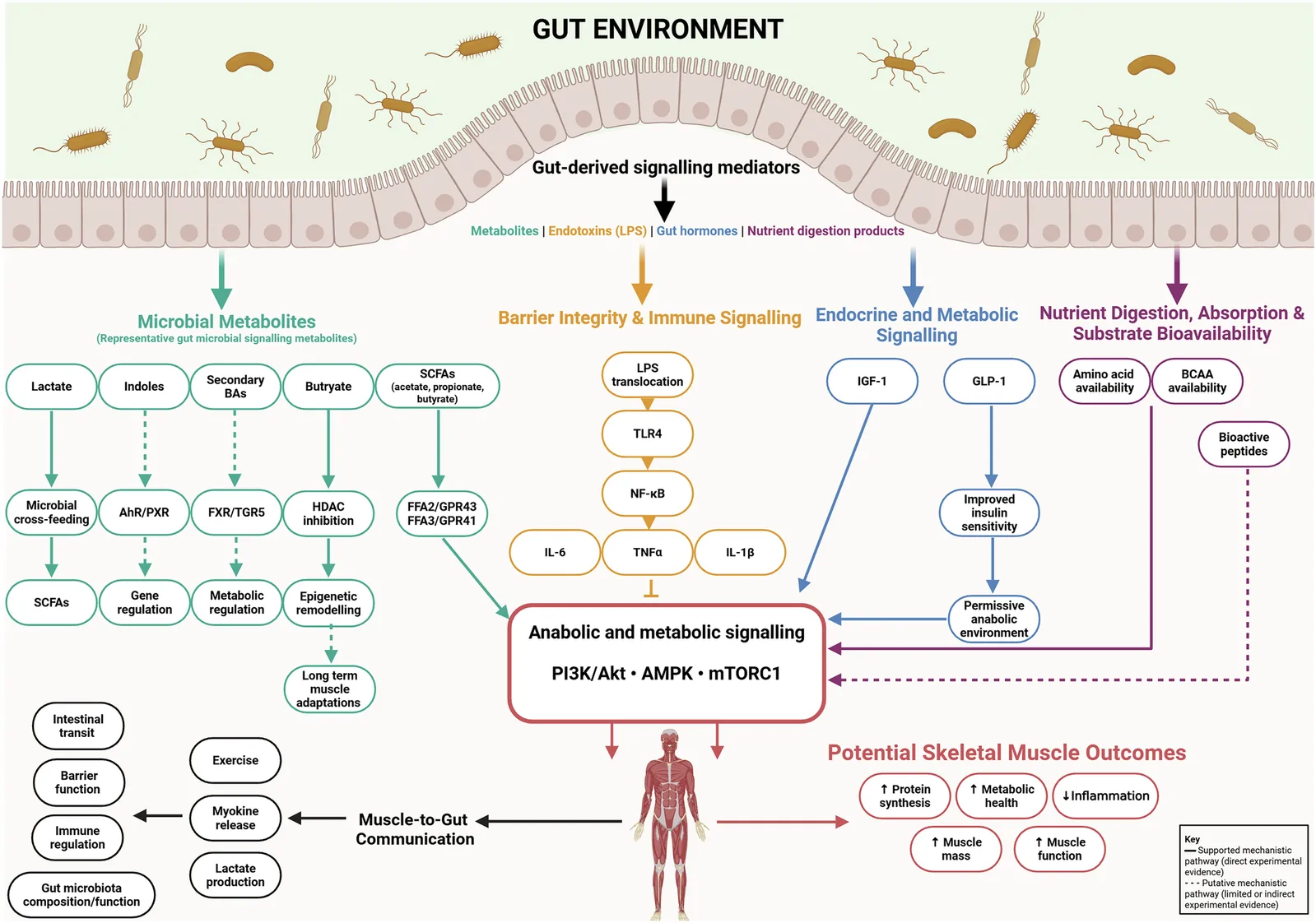
Overview of the gut-muscle axis and principal pathways linking the gut microbiome with skeletal muscle physiology. The gut microbiome influences skeletal muscle through multiple interconnected mechanisms involving microbial metabolite production, intestinal barrier integrity, immune regulation, endocrine signalling and nutrient metabolism. Representative gut microbial signalling metabolites, including short-chain fatty acids (SCFAs), secondary bile acids, indole derivatives and lactate, interact with host receptors and intracellular signalling pathways to influence anabolic responsiveness, inflammation and cellular metabolism. These signals converge on canonical intracellular pathways involved in skeletal muscle regulation, including PI3K/Akt, AMPK and mTORC1, thereby influencing muscle protein synthesis (MPS), metabolic function and muscle adaptation. Maintenance of intestinal barrier integrity limits translocation of bacterial products such as lipopolysaccharide (LPS), thereby reducing activation of inflammatory pathways including TLR4–NF-κB signalling and downstream cytokine production (e.g. TNF-α, IL-1β and IL-6). In parallel, gut-derived endocrine mediators (e.g. GLP-1 and IGF-1) together with improvements in insulin sensitivity and enhanced nutrient digestion and amino acid bioavailability may further support an anabolic environment conducive to MPS. Communication within the gut-muscle axis is bidirectional, with skeletal muscle influencing gut microbial ecology through exercise, myokine release and lactate production, thereby contributing to reciprocal host–microbiome interactions that regulate whole-body metabolic homeostasis. Solid arrows indicate mechanistic pathways supported by experimental evidence, whereas dashed arrows denote emerging or putative mechanisms for which direct evidence linking gut-derived signalling to skeletal muscle physiology remains limited, particularly in humans.

The GMA describes the bidirectional communication network between the gastrointestinal tract, its resident microbiome, and skeletal muscle through interconnected metabolic, immune, endocrine and neural signalling pathways (Fig. 1). The GMA is a dynamic signalling network in which gut-derived metabolites, microbial structural components, host immune mediators, nutrient availability and endocrine factors converge to regulate skeletal muscle metabolism, mitochondrial function, protein turnover and physical performance^21–23^. Conversely, skeletal muscle influences gut physiology through exercise-induced alterations in intestinal transit, barrier function, immune regulation and microbial composition^24–26^, highlighting the reciprocal nature of this axis. Consequently, understanding the mechanisms underpinning gut-muscle communication requires consideration of multiple interacting biological pathways rather than isolated signalling events.

Current evidence suggests that communication within the GMA occurs predominantly through four interrelated mechanistic domains: (1) microbial metabolites produced during fermentation of dietary substrates, particularly short-chain fatty acids (SCFAs); (2) modulation of intestinal barrier integrity and systemic immune signalling; (3) endocrine and metabolic communication involving gut-derived hormones and host anabolic signalling pathways; and (4) alterations in nutrient digestion, absorption and substrate availability. While these pathways are discussed separately for clarity, considerable crosstalk exists, with many converging on common intracellular regulators of skeletal muscle metabolism such as AMP-activated protein kinase (AMPK), phosphoinositide 3-kinase (PI3K)/Akt, and mechanistic target of rapamycin complex 1 (mTORC1).

### Microbial metabolites

Among gut-derived metabolites, SCFAs arguably represent the best characterised mechanistic link between the gut microbiome and skeletal muscle physiology. Produced predominantly through bacterial fermentation of non-digestible carbohydrates, and to a lesser extent through fermentation-associated dietary substrates, the prominent SCFAs, acetate, propionate and butyrate, exert pleiotropic effects on host physiology through both receptor-dependent and receptor-independent mechanisms. Importantly, these mechanisms extend beyond local effects within the intestine and include modulation of systemic inflammation, endocrine signalling, mitochondrial metabolism and intracellular pathways regulating skeletal muscle protein turnover.

Following intestinal production, SCFAs are absorbed by colonocytes and enter circulation, where they can influence peripheral tissues including skeletal muscle either directly or indirectly through endocrine and immune-mediated pathways^27,28^. Receptor-dependent signalling is principally mediated via the G-protein-coupled receptors free fatty acid receptor 2 (FFA2/GPR43) and free fatty acid receptor 3 (FFA3/GPR41), which exhibit differing affinities for acetate, propionate and butyrate and activate distinct downstream signalling cascades^29–31^. While these receptors are most abundantly expressed within intestinal epithelial cells and immune tissues, evidence suggests that receptor-mediated signalling contributes to systemic metabolic regulation through effects on insulin sensitivity, substrate utilisation and inflammatory tone^28^, thereby indirectly influencing skeletal muscle physiology. In preclinical models, butyrate has additionally been shown to activate PI3K/Akt signalling through FFA2, promoting downstream mTORC1 activity and attenuating skeletal muscle atrophy under diabetic conditions^32^.

In addition to receptor-mediated signalling, butyrate possesses receptor-independent biological activity through inhibition of histone deacetylases (HDACs), thereby altering chromatin accessibility and gene transcription^33^. This epigenetic mechanism has been implicated in the regulation of genes involved in mitochondrial biogenesis, oxidative metabolism, antioxidant defence and inflammatory signalling^33,34^, representing a route through which microbial metabolites may influence longer-term skeletal muscle adaptation. Consequently, the biological effects of butyrate may extend beyond acute metabolic regulation to include potential epigenetic remodelling of skeletal muscle phenotype. Although this mechanism is well-established in broader SCFA biology, its relevance to human skeletal muscle adaptation following dietary modulation of the gut microbiome remains incompletely characterised.

Collectively, these findings illustrate that SCFAs have the capacity to influence multiple intracellular signalling pathways central to skeletal muscle metabolism. Among these, AMPK and mTORC1 represent complementary regulators of cellular energy sensing and anabolic activity. Activation of AMPK promotes mitochondrial biogenesis, fatty acid oxidation and metabolic flexibility during energetic stress^35^, whereas mTORC1 stimulates protein synthesis and muscle hypertrophy under nutrient-replete conditions^36–38^. Rather than indicating conflicting biology, these findings likely reflect the context-dependent nature of butyrate signalling and highlight that AMPK and mTOR should be viewed as components of an integrated metabolic regulatory network rather than independent pathways.

SCFAs likely represent only one component of a much broader metabolite signalling network. These include secondary bile acids, lactate, as well as indole derivatives arising from microbial tryptophan metabolism.

Gut microbial metabolism generates secondary bile acids through bacterial transformation of primary bile acids. These metabolites act as signalling molecules through receptors including the farnesoid X receptor (FXR) and Takeda G-protein-coupled receptor 5 (TGR5)^39–41^, with TGR5 notably abundant in skeletal muscle and brown adipose tissue. FXR-mediated signalling leads to downstream regulation of genes involved in bile acid, glucose, and lipid metabolism, while TGR5-mediated signalling operates via pathways with subsequent enhancement of energy expenditure and glucose homeostasis^42^. These signalling cascades integrate with other metabolic pathways, including insulin signalling, mitochondrial function regulation, and modulation of inflammatory responses^43,44^, which hold relevance to skeletal muscle.

Microbial metabolism of dietary tryptophan generates a diverse range of indole derivatives, including indole-3-acetate, indole-3-propionate and indole-3-aldehyde, which signal through the aryl hydrocarbon receptor (AhR) and pregnane X receptor (PXR)^45^. Activation of these pathways contributes to maintenance of intestinal barrier integrity, regulation of mucosal immune responses and attenuation of systemic inflammation, thereby potentially reducing inflammatory impingement on anabolic pathways^46^.

Traditionally regarded as a by-product of anaerobic metabolism, lactate is increasingly recognised as an important signalling metabolite between skeletal muscle and the gut. Exercise-induced lactate can alter gut microbial composition by promoting the growth of lactate-utilising bacteria, promoting bacterial cross-feeding, and converting lactate into other SCFAs. An example was demonstrated by Scheiman and colleagues, who found that *Veillonella atypica* mediated lactate-to-propionate conversion enhanced treadmill performance in a mouse model^47^. Microbial lactate production may also contribute to host metabolic signalling through effects on substrate utilisation, immune regulation and inter-organ metabolic communication^48^. These reciprocal interactions suggest that lactate may represent a significant mediator of bidirectional gut-muscle crosstalk, although direct evidence demonstrating functional consequences for skeletal muscle adaptation remains limited.

It is important to reiterate that this mechanistic evidence largely stems from preclinical models; however, these findings indicate that a diverse repertoire of microbial metabolites may have the capacity to influence skeletal muscle not only through direct metabolic signalling, but also by shaping the inflammatory and endocrine environment in which muscle adaptation occurs. Consequently, maintenance of intestinal barrier integrity and regulation of systemic immune signalling represent a second major mechanistic domain of the GMA.

### Intestinal barrier integrity and immune signalling

Beyond direct metabolic signalling, the gut microbiome can influence skeletal muscle through regulation of intestinal barrier integrity and systemic immune homeostasis. The intestinal epithelium forms a highly selective barrier that permits nutrient absorption while restricting translocation of luminal microorganisms, endotoxins and other pro-inflammatory molecules into the systemic circulation^27,28^. This barrier is maintained through coordinated interactions between epithelial cells, mucus layers, tight junction proteins, immune cells and the resident gut microbiome^28^. Disruption of this homeostatic environment can culminate in impaired intestinal barrier function and increased intestinal permeability, facilitating the passage of bacterial products such as lipopolysaccharide (LPS) into the circulation, promoting chronic low-grade inflammation^27^.

Systemic exposure to LPS activates innate immune pathways, principally through Toll-like receptor 4 (TLR4)-mediated activation of nuclear factor-κB (NF-κB), resulting in increased production of pro-inflammatory cytokines including tumour necrosis factor-α (TNF-α), interleukin-6 (IL-6) and interleukin-1β (IL-1β)^49,50^. Although transient inflammatory responses are essential for immune defence and tissue repair, persistent low-grade inflammation has been implicated in the pathogenesis of sarcopenia, obesity and other chronic metabolic disorders characterised by impaired skeletal muscle function^51^. Importantly, butyrate may contribute to preservation of barrier integrity through maintenance of epithelial energy metabolism and tight junction function^28,33^, thereby indirectly reducing systemic inflammatory burden.

These inflammatory pathways have important implications for skeletal muscle anabolism. Chronic elevation of pro-inflammatory cytokines has been shown to impair insulin signalling^49^, suppress PI3K/Akt/mTOR-mediated protein synthesis^49,51^, and promote activation of proteolytic pathways^51^, which could contribute to the development of anabolic resistance during ageing and chronic disease.

### Endocrine and metabolic signalling

Beyond regulation of immune homeostasis, the gut microbiome may have the capacity to modulate host endocrine and metabolic signalling, providing an additional pathway through which microbial activity could influence skeletal muscle metabolism. Communication between the gut microbiome and the endocrine system occurs through multiple mechanisms, including modulation of gut-derived hormones, insulin sensitivity, nutrient sensing and circulating growth factors^27,28^. Rather than acting independently, these endocrine responses interact closely with microbial metabolites and inflammatory pathways, converging on intracellular regulators of muscle protein turnover such as PI3K/Akt and mTORC1.

Among these endocrine mediators, insulin-like growth factor-1 (IGF-1) has received attention owing to its central role in regulating skeletal muscle growth, protein synthesis and regeneration^52,53^. Experimental studies demonstrate that germ-free animals exhibit reduced circulating IGF-1 concentrations, impaired skeletal muscle development and altered anabolic signalling, with partial restoration following microbial colonisation or microbiome transfer^54^. This regulation may be partly tied to the availability of de novo microbially synthesised branched-chain amino acids (BCAAs) or the generation of catabolic derivatives. For instance, specific structural components of the gut microbiome, such as the *ilvBN* and *ilvE* gene clusters found in taxa like *Prevotella copri*, govern de novo BCAA synthesis, influencing the systemic pool that cross-talks with host growth factors^55,56^.

Similarly, antibiotic-induced disruption of the gut microbiome has been associated with reductions in muscle mass and impaired anabolic signalling in preclinical models^22^, further supporting a role for the gut microbiome in maintenance of skeletal muscle phenotype. These findings suggest that the gut microbiome can modulate IGF-1 signalling under specific experimental conditions; however, they do not indicate that the microbiome is the primary regulator of systemic IGF-1, which remains predominantly governed by host endocrine physiology^53,57^. In this context, evidence connecting microbiome composition directly to circulating IGF-1 concentrations and muscle anabolism in humans remains comparatively limited^11,21^, with most current data derived from animal models.

The gut microbiome may additionally influence anabolic signalling through improvements in insulin sensitivity and glucose homeostasis, thereby enhancing the efficiency with which skeletal muscle responds to nutrient intake^27,34,58^. Gut-derived hormones, including glucagon-like peptide-1 (GLP-1), may contribute to these metabolic effects through regulation of glucose homeostasis, insulin secretion and insulin sensitivity^59^. This endocrine axis can be modulated by microbial BCAA catabolism; specialised commensals including *Parabacteroides merdae*, *Faecalibacterium prausnitzii*, and specific *Butyrivibrio* species utilise branched-chain amino acid aminotransferases (BCAT) and branched-chain α-keto acid dehydrogenase (BCKDH) enzyme complexes to degrade luminal leucine, isoleucine, and valine^60^. This degradation pathway bypasses systemic accumulation and instead yields branched-chain fatty acids (BCFAs), specifically isobutyrate, isovalerate, and 2-methylbutyrate^60,61^. These BCFAs act as signalling ligands that bind to GPR41 and GPR43 on colonic enteroendocrine L-cells, working in synergy with SCFAs to amplify the local secretion of endogenous GLP-1^62^. However, direct evidence demonstrating that GLP-1 independently stimulates MPS remains limited, suggesting that its contribution to the GMA is more likely to be indirect rather than via direct anabolic signalling.

### Nutrient digestion, amino acid availability and anabolic substrate delivery

In addition to the mechanisms highlighted, the gut microbiome may influence skeletal muscle by altering the digestion, absorption and metabolic fate of dietary nutrients. Protein digestion and amino acid availability are fundamental determinants of MPS, yet these processes are increasingly recognised as being influenced by interactions between dietary substrates, digestive physiology and the gut microbiome. Microbial metabolism can modify the availability of amino acids reaching the distal intestine, generate bioactive metabolites from dietary proteins and influence intestinal nutrient absorption, thereby shaping the anabolic environment in which skeletal muscle responds to feeding^27,28^.

BCAAs, particularly leucine, play a central role in activation of mTORC1 and stimulation of MPS^63,64^. However, the gut microbiome also actively participates in amino acid metabolism through both biosynthetic and catabolic pathways, meaning that microbial activity may influence the quantity, form and temporal availability of amino acids reaching the host^65^, which could potentiate nutrient-sensitive anabolic pathways such as mTORC1. The net physiological effect will likely depend on the balance within the microbial ecology between BCAA producers and consumers. For instance, an overabundance of BCAA-synthesising taxa (e.g., *Prevotella copri* or *Phocaeicola vulgatus*) may lead to excessive systemic BCAA accumulation, which paradoxically promotes insulin resistance and anabolic blunting in myocytes^66^. Conversely, active BCAA-degrading taxa (such as *Eubacterium siraeum*, *Ruminococcus gnavus*, and specific proteolytic *Clostridium* or *Bacteroides* species) utilise transamination pathways to process excess free luminal BCAAs, regulating the systemic amino acid curve and preventing metabolic stagnation^67^.

Furthermore, it is important to highlight that microbial fermentation of undigested dietary proteins also generates phenolic compounds such as p-cresol, as well as indoles, ammonia and hydrogen sulphide associated with impaired barrier integrity, inflammation, metabolic dysfunction and colorectal cancer carcinogenesis^27,68^. The physiological consequences of the generation of these metabolites will likely depend on substrate availability and microbial ecology. For example, fermentation of protein within a fibre-rich dietary environment may support production of beneficial signalling metabolites, whereas excessive proteolytic fermentation in low-fibre diets may consequently culminate in an undesirable proteolytic environment with a greater presence of detrimental metabolites^69–71^.

## Translation of mechanistic basis of the gut-muscle axis in humans

Although substantial mechanistic and preclinical evidence supports the existence of the GMA, translation of these mechanisms to human physiology remains comparatively limited.

Observational studies have associated microbial diversity and specific taxa with measures of frailty, physical function, and muscle mass in older adults^72,73^, while athletes and highly trained individuals have been reported to exhibit distinct microbiome profiles compared with sedentary controls, raising the possibility that microbial ecology interacts with physical activity to influence muscle adaptation and recovery^25,47,74^. However, while observational data can be informative, it cannot establish causality, and it is important to evaluate causal evidence from randomised controlled trials (RCTs). In this context, intervention studies manipulating the microbiome through probiotics or dietary fibre have demonstrated effects on inflammatory markers, insulin sensitivity, and metabolic flexibility, factors related to muscle protein metabolism^58,75^. However, due to the lack of direct assessments of skeletal muscle endpoints, the question remains as to whether these systemic changes translate into meaningful improvements in skeletal muscle biology.

An additional consideration is whether gut microbiome-derived signals exert equivalent effects across tissues or whether skeletal muscle represents a uniquely responsive target. While SCFAs, bile acids, indole derivatives and inflammatory mediators influence multiple organs, including adipose tissue, liver, cardiac muscle and the central nervous system^28^, skeletal muscle possesses several characteristics that may confer heightened responsiveness to nutritional and microbial cues. As the largest metabolic organ in the body, accounting for approximately 40% of total body mass^76^, skeletal muscle exhibits remarkable metabolic plasticity and continuously remodels in response to nutrient availability, physical activity and endocrine signalling^77^. Consequently, microbial influences on insulin sensitivity, mitochondrial function, inflammatory tone and amino acid availability may have particularly important implications for muscle protein turnover and functional adaptation. In contrast, cardiac and smooth muscle tissues are governed by distinct physiological priorities related to continuous contractile function and homeostatic regulation^78^, suggesting that microbiome-derived signals may produce tissue-specific outcomes. Understanding these differential responses will be important for defining whether dietary strategies targeting the gut microbiome can selectively enhance skeletal muscle health.

Consequently, although mechanistic and preclinical evidence support a role of GMA in skeletal muscle regulation, causal pathways linking microbiome modulation to muscle-specific outcomes in humans remain incompletely characterised, and limitations currently restrict mechanistic interpretation of human studies. First, most studies rely on systemic biomarkers, with direct assessment of skeletal muscle phenotypes in humans less frequently undertaken, likely due to the invasiveness of techniques such as biopsy as well as the need for specialist facilities and personnel to perform such investigations. Second, microbial composition is often reported without functional or metabolomic characterisation, hindering interpretation of mechanistic pathways. Third, the interactive effects of dietary protein quality, microbiome composition, and anabolic responsiveness are rarely examined simultaneously. These limitations are particularly relevant when considering FPFs, which uniquely combine bioavailable protein with microbial and fermentation-derived bioactive components which carry the potential to modulate the GMA.

## Fermented protein foods: microbial, nutritional, and bioactive profiles

**Fig. 2:**
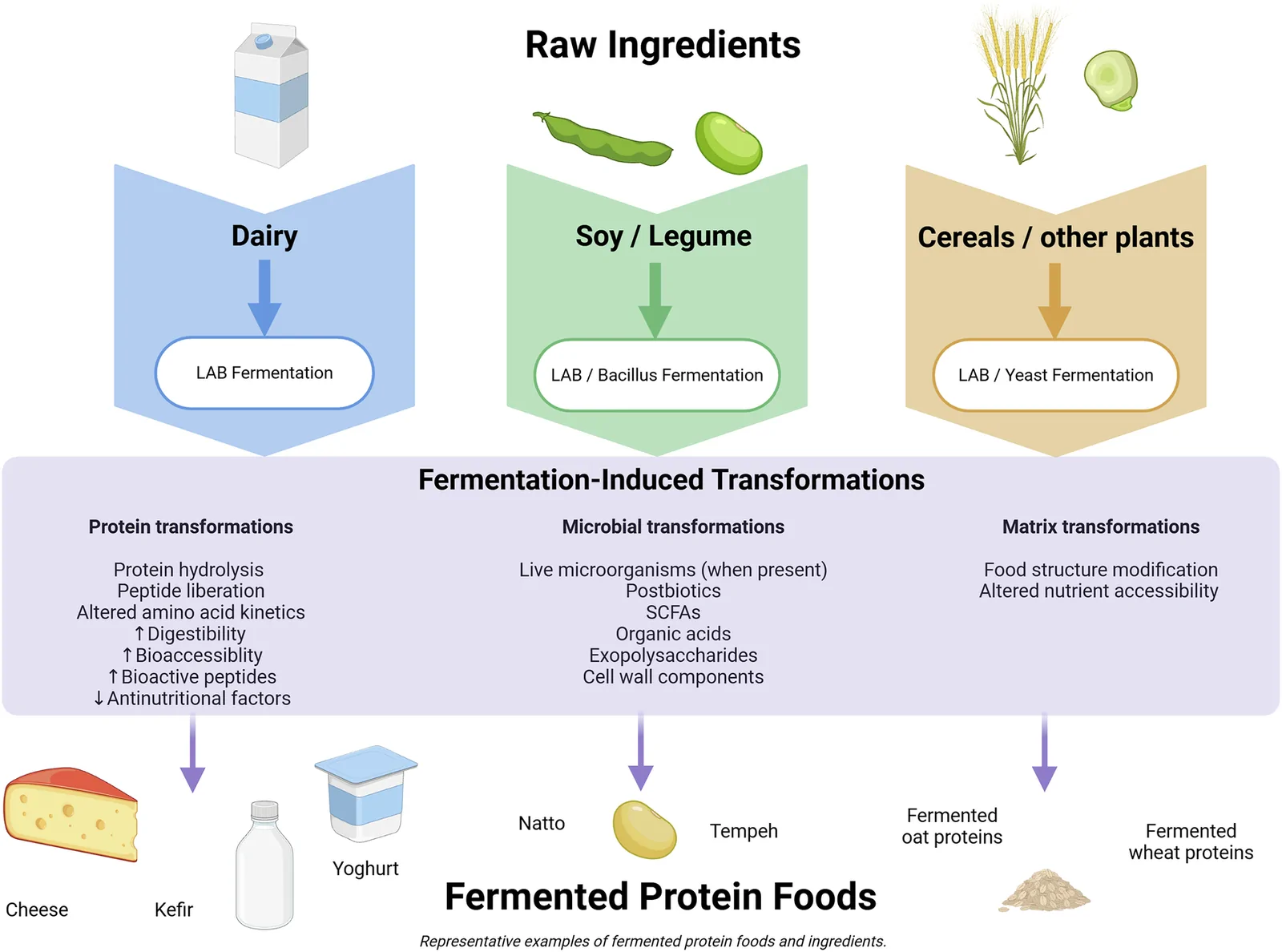
Fermentation-induced transformations that distinguish fermented protein foods from their raw protein ingredients. Raw protein sources from dairy, soy/legumes and cereal or other plant matrices undergo microbial fermentation, resulting in coordinated modifications to the protein substrate, microbial composition and food matrix. Protein transformations include hydrolysis, liberation of bioactive peptides, altered amino acid kinetics, improved digestibility and bioaccessibility, and reduced concentrations of selected antinutritional factors. Concurrently, microbial activity generates live microorganisms (when present), microbial components, postbiotics, short-chain fatty acids (SCFAs), organic acids, exopolysaccharides and other fermentation-derived metabolites. Fermentation also modifies the physical structure of the food matrix, influencing nutrient accessibility and gastrointestinal digestion. Collectively, these fermentation-induced transformations produce FPFs that differ from their unfermented counterparts in both composition and functional properties, providing a mechanistic basis for investigating their potential effects on host physiology, including the gut-muscle axis. Representative examples of fermented protein foods are shown beneath each substrate category.

FPFs represent a diverse category of dietary products in which microbial activity transforms the native protein matrix, generating bioactive compounds with potential implications for human health. Traditional dairy-based FFs, including yoghurt, kefir, and cheeses, have been widely studied^79^, while plant-based alternatives, such as fermented soy, pea, wheat and oat protein products, are emerging in the market and scientific literature^80–83^. Rather than acting solely as a preservation process, fermentation alters the physicochemical properties of protein-containing foods through microbial metabolism. These changes extend beyond the introduction of viable microorganisms to include protein hydrolysis, liberation of bioactive peptides, production of microbial metabolites and modification of the food matrix, creating a complex nutritional system (Fig. 2).

The microbial communities responsible for fermentation are dominated by LAB (including Lactobacilli, *Lactococcus*, *Streptococcus* and *Leuconostoc)*, which drive acidification and flavour development. These microbes possess enzymatic capacities that drive protein hydrolysis, carbohydrate fermentation, and bioactive metabolite synthesis^19^. Microbially derived proteases partially hydrolyse intact proteins into smaller peptides and free amino acids, increasing protein solubility and potentially improving digestibility^18,19^. For the most part, fermentation does not substantially alter the intrinsic amino acid composition of dietary proteins; rather, it may enhance the liberation and postprandial availability of indispensable amino acids through modification of protein structure^18,80^. These effects may be particularly relevant for plant-derived proteins, where fermentation can additionally reduce antinutritional factors such as phytates and protease inhibitors, thereby improving amino acid bioaccessibility and overall protein quality. Consequently, fermentation may improve functional measures of protein quality^80,81^, including digestibility and Digestible Indispensable Amino Acid Scores (DIAAS), particularly in plant-based protein foods.

Proteolysis during fermentation additionally liberates numerous peptide sequences that exhibit antioxidant, angiotensin-converting enzyme (ACE)-inhibitory, anti-inflammatory and immunomodulatory activities in vitro^17,18^. These bioactive peptides represent one potential mechanism through which FPFs may influence host physiology independently of their amino acid content. However, although biological activities of many peptide sequences have been demonstrated under experimental conditions, their physiological relevance remains incompletely established. Some peptides are further hydrolysed during gastrointestinal digestion, and relatively few have been shown to survive intestinal transit, enter the circulation intact, or reach peripheral tissues at concentrations sufficient to exert biological effects in vivo^18^.

Beyond protein hydrolysis, microbial taxa produce metabolites such as lactic acid and SCFAs; with their relevance in gut-muscle communication indicated above, while exopolysaccharides may also be produced, which can influence host immune pathways, reducing inflammation and strengthening the gut barrier^84,85^. Depending on the food, FPFs may also contain viable microorganisms, non-viable microbial components (postbiotics) and fermentation-derived metabolites capable of interacting with the resident gut microbiome following consumption^15,16^. The relative abundance of these constituents will vary considerably according to starter culture selection, fermentation duration, substrate type and subsequent processing and storage conditions.

In addition to modifying protein chemistry and microbiome composition, fermentation alters the physical structure of the food matrix itself. Changes in pH, protein conformation, nutrient accessibility and matrix architecture may influence gastric emptying, digestive kinetics and nutrient absorption^18,80^. As a result, the biological properties of FPFs are unlikely to reflect any single fermentation product but instead arise from interactions between altered protein structure, microbial metabolites, liberated peptides, viable microorganisms and/or components thereof, and the surrounding food matrix.

Taken together, FPFs represent complex nutritional matrices in which improved protein bioavailability, fermentation-derived peptides, microbial metabolites and food matrix modifications may act synergistically to influence host physiology. These integrated characteristics provide the biological rationale for considering FPFs as potential modulators of the GMA and establish the mechanistic foundation for the pathways discussed in the following section.

## Potential mechanistic pathways where fermented protein foods could impact skeletal muscle

**Fig. 3:**
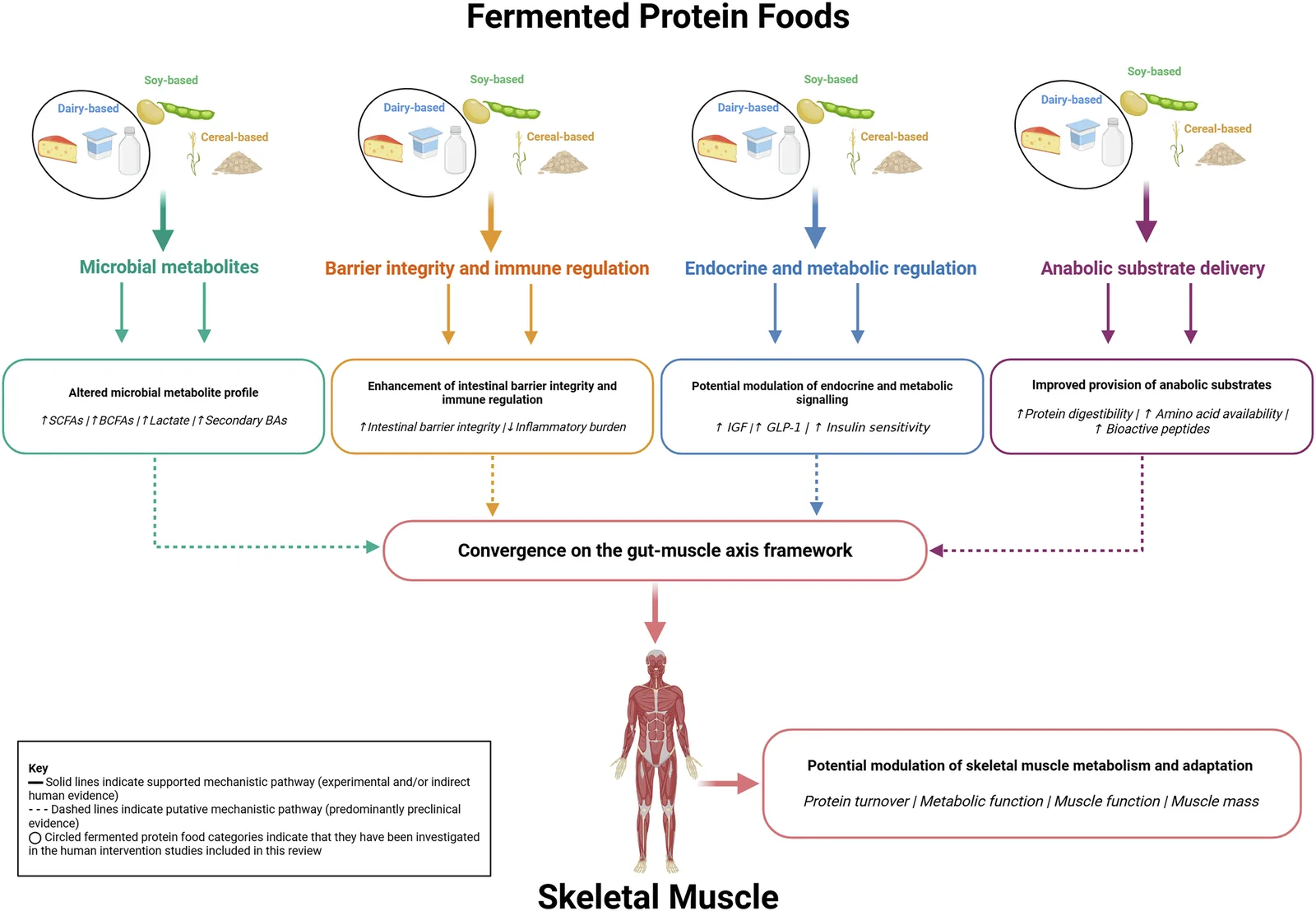
Proposed mechanisms through which fermented protein foods may influence skeletal muscle physiology via the gut-muscle axis. Fermented protein foods (FPFs) may influence skeletal muscle through multiple complementary pathways. Four principal mechanistic domains are illustrated^1^: modulation of microbial metabolite production, including short-chain fatty acids (SCFAs), secondary bile acids, indole derivatives and lactate^2^; enhancement of intestinal barrier integrity and attenuation of inflammatory signalling^3^; modulation of endocrine and metabolic pathways, including GLP-1 and IGF-1; and^4^ enhanced protein quality and anabolic substrate delivery through improved protein digestibility, amino acid bioavailability and fermentation-derived bioactive peptides. These mechanisms are illustrated as feeding directly into a centralised “Convergence on the gut-muscle axis framework,” with the downstream intracellular signalling cascades (e.g., PI3K/Akt, AMPK, and mTORC1) detailed in Fig. 1.Solid arrows denote localised mechanistic pathways supported by direct experimental and/or indirect human evidence. Notably, all four primary mechanistic domains transition to the central convergence box via dashed arrows; this highlights that while these intermediate food-derived signals are well-established, evidence directly demonstrating that these upstream mechanisms culminate in clear physiological impacts on skeletal muscle remains putative or supported predominantly by preclinical models. Furthermore, circled fermented protein food categories indicate those for which human intervention evidence is currently available within the literature reviewed, emphasising that active human evidence is presently concentrated in dairy-based FPFs and remains critically limited for plant-derived alternatives.

FPFs have the capacity to influence skeletal muscle through multiple, interrelated mechanisms that correspond closely with the four mechanistic domains of the GMA described earlier. Fermentation modifies the nutritional and microbial characteristics of dietary protein in ways that may influence microbial metabolite production, intestinal barrier function, endocrine and metabolic signalling, as well as nutrient availability^15,16^ (Fig. 3). These mechanisms provide a biologically plausible framework through which FPFs could modulate skeletal muscle physiology beyond dietary protein.

### Modulation of microbial metabolites

FPFs deliver fermentation-derived metabolites directly and, depending on the food, viable microorganisms capable of interacting with the resident gut microbiome. Consumption of FPFs may therefore modify both microbial composition and, pertinently, the repertoire of metabolites produced following fermentation within the gastrointestinal tract. These include SCFAs, lactate, indoles, secondary bile acids amongst other potentially bioactive compounds, which, as previously outlined, have been implicated in regulation of biological pathways and processes with relevance to skeletal muscle physiology^16,27,28,41,45^.

### Intestinal barrier integrity and immune regulation

Fermentation-derived metabolites, including SCFAs, exopolysaccharides and microbial cell components, may additionally support intestinal barrier integrity and immune homeostasis. Experimental evidence suggests that these compounds can strengthen epithelial barrier function, reduce endotoxin translocation and attenuate activation of pro-inflammatory pathways involving TLR4 and NF-κB^28,32,49–51^. By lowering chronic low-grade inflammation, FPFs may indirectly reduce catabolic signalling within skeletal muscle and create a more permissive environment for MPS. Consistent with this concept, systematic reviews of fermented dairy interventions report reductions in circulating inflammatory biomarkers including CRP, TNF-α and IL-6^86–88^.

### Endocrine and metabolic regulation

While the protein fraction of FPFs can stimulate established anabolic pathways through essential acid provision, modulating mediators such as IGF-1 and mTORC1^64^, fermentation-derived metabolites may additionally influence host metabolic regulation through improvements in insulin sensitivity, glucose homeostasis and nutrient partitioning, which may enhance the anabolic milieu. SCFAs acting on GPR43-mediated signalling in adipocytes and hepatocytes may stimulate insulin signalling pathways, leading to increased glucose uptake by these cells and potentially improving overall insulin sensitivity^89^. In this context, human studies of fermented dairy products report improvements in insulin sensitivity and glycaemic control^58,90–93^. In addition, SCFAs act as secretagogues for gut-derived hormones such as GLP-1^94^, which can enhance postprandial insulin secretion and nutrient handling^59^. Improved insulin action may also facilitate amino acid delivery to skeletal muscle and suppress muscle protein breakdown^95,96^. Taken together, such scenarios have the potential of creating a more permissive environment for skeletal muscle anabolism. Nevertheless, direct evidence demonstrating that FPFs increase circulating anabolic hormones such as IGF-1 or directly stimulate MPS through endocrine pathways is currently lacking. Consequently, endocrine effects should be regarded as indirect facilitators of an anabolic milieu rather than primary drivers of skeletal muscle adaptation.

### Protein quality and anabolic substrate delivery

As described earlier, fermentation modifies the physicochemical properties of dietary proteins through partial hydrolysis, increasing digestibility and altering postprandial amino acid kinetics. Although fermentation generally does not substantially alter the intrinsic amino acid composition of dietary proteins, it may improve protein digestibility and amino acid bioaccessibility through modification of protein structure and reduction of antinutritional factors, particularly pertinent for plant-derived protein foods^18,80,81^. Consequently, the rate and efficiency of leucine and other indispensable amino acid delivery to skeletal muscle may be enhanced, which could facilitate activation of mTORC1 and MPS^64^. Fermentation additionally liberates numerous bioactive peptides; however, as discussed previously, the physiological relevance of many peptides remains uncertain because oral bioavailability and systemic exposure appear limited^18^. Microbial metabolism may also utilise amino acids, including BCAAs, under certain conditions, indicating that fermentation should not be viewed as uniformly enhancing anabolic potential^65^. Rather, the net effects on amino acid availability are likely to depend upon substrate characteristics, fermentation conditions and microbial taxa involved.

Collectively, the characteristics of FPFs indicate that they represent complex nutritional matrices capable of interacting with multiple components of the GMA. Importantly, as will be highlighted in the following section, many of these mechanisms remain biologically plausible rather than clinically established, and considerable uncertainty persists regarding the relative contribution of microbial metabolites, bioactive peptides, improvements in protein quality and alterations in host metabolism to observed effects on skeletal muscle.

## Clinical evidence for fermented protein food modulation of skeletal muscle physiology

Although the mechanistic rationale for FPF modulation of skeletal muscle physiology is biologically plausible and promising, direct clinical evidence demonstrating improvements in muscle-specific outcomes remains limited. Existing RCTs vary considerably with respect to study population, FPF matrix, protein dose, comparator, intervention duration and outcome measures, limiting direct comparison across studies. Due to this, current evidence should be interpreted as providing varying levels of support across different mechanistic components of the GMA, rather than definitive proof of causal efficacy. The characteristics of these studies are provided in Table 1 and Table 2 and are elaborated below.

**Table 1:** Human studies investigating fermented protein foods and reporting direct skeletal muscle outcomes

Study	Design	Population	Intervention	Comparator	Duration	Primary outcome	Muscle-relevant outcomes	Microbiome data	Main statistically significant findings	Interpretation
Kim et al. 2023^97^	DB- PG-RCT	Middle-aged healthy adults (*n* = 48, mean age 44.8 yrs)	Fermented whey protein (*Lacticaseibacillus* casei DK211) ~37 g, twice daily	Non-fermented whey protein	8 weeks	Muscle strength, physical performance	Grip strength, lean mass, functional performance	Not reported	Statistically significant between-group improvement in dynamic balance only (both limbs; two-way ANOVA *p* = 0.032). Grip strength, lean mass and back strength improved within the fermented group, but between-group differences were not statistically significant.	Modest direct evidence for benefit, although effects were limited and the absence of microbiome or mechanistic data precludes attribution specifically to fermentation.
Jung et al. 2025^98^	PC- DB- PG-RCT	Healthy adults (*n* = 53, mean age 54.5 yrs)	Postbiotic-fermented protein beverage prepared from *Lentilactobacillus kefiri* DH5 postbiotics + whey protein ( ~ 6 g/day)	Non-fermented beverage	12 weeks	Muscle strength, gut microbiome composition	Handgrip strength, SCFA levels	Gut microbiome analysis; increased beneficial taxa and SCFA producers	No statistically significant between-group improvements were observed for the primary outcome (dominant-hand grip strength; ITT *p* = 0.498; PP *p* = 0.363). Within the KP group, dominant-hand grip strength increased statistically significantly from baseline (ITT *p* = 0.012; PP *p* = 0.025). Lean mass, IGF-1 and body composition were unchanged. KP supplementation reduced IL-1β (within-group *p* = 0.011), increased irisin (within-group *p* = 0.027), and altered gut microbiome composition, including increases in *Faecalibacterium* and *Latilactobacillus*.	Findings were hypothesis-generating, with no superiority for the primary endpoint and limited generalisability because the intervention was a postbiotic beverage rather than an intact FPF.
Iwasa et al. 2013^91^	PC- CO-RCT	Young healthy men (*n* = 18, mean age 21.6 yrs)	*Lactobacillus* helveticus-fermented milk (600 mL/day of fermented milk (providing approximately 6.6 g protein))	Non-fermented milk	Single bout/24–48 h	Exercise recovery, soreness	Muscle soreness, CK levels	Not assessed	Fermented milk statistically significantly reduced total post-exercise muscle soreness compared with placebo (12.6 ± 1.1 vs 14.2 ± 1.2; *p* < 0.05) and prevented the exercise-induced reduction in respiratory quotient and carbohydrate oxidation (*p* < 0.05). Serum creatine phosphokinase showed a non-significant trend towards reduction.	Suggests potential benefits for acute exercise recovery, but the small sample, short follow-up and absence of microbiome or long-term muscle outcomes limit interpretation.

*ANOVA* analysis of variance, *CK* creatine kinase, *CO-RCT* crossover randomised controlled trial, *DB* double-blind, *FPF* fermented protein food, *IGF-1* insulin-like growth factor-1, *IL-1β* interleukin-1 beta, *ITT* intention-to-treat, *KP* kefir postbiotic, *PC* placebo-controlled, *PG-RCT* parallel-group randomised controlled trial, *PP* per-protocol, *SCFA* short-chain fatty acid.

**Table 2:** Human studies investigating fermented protein foods reporting indirect muscle-relevant immunometabolic or microbiome outcomes

Study	Design	Population	Intervention	Comparator	Duration	Primary outcome	Muscle-relevant outcomes	Microbiome data	Main statistically significant findings	Interpretation
Ostadrahimi et al. 2015^92^	PC- DB- PG-RCT	Adults with type 2 diabetes (*n* = 60, mean ages 35–65 yrs)	Kefir (600 mL/day, containing *Streptococcus thermophiles*, enriched with *Lacticaseibacillus casei*, *Lactobacillus acidophilus* and *Bifidobacterium lactis.)*	Standard fermented milk (containing *Streptococcus thermophilus and Lactobacillus bulgaricus)*	8 weeks	Glucose (glycated haemoglobin (HbA1c)) and lipid profile (low-density lipoprotein cholesterol (LDL-C)) and high-density lipoprotein cholesterol (HDL-C)	Insulin sensitivity (HOMA-IR), fasting glucose	Not assessed	Daily consumption of 600 mL/day probiotic kefir for 8 weeks significantly improved glycaemic control compared with conventional fermented milk. HbA1c decreased within the kefir group (*p* = 0.001) and remained significantly lower than the control following ANCOVA adjustment (*p* = 0.02). Fasting blood glucose was also significantly lower than control after intervention (adjusted *p* = 0.03). No significant between-group differences were observed for triglycerides, total cholesterol, LDL-C or HDL-C.	Improved glycaemic outcomes, but the absence of muscle, microbiome and inflammatory measurements limits relevance to gut-muscle mechanisms.
Bellikci-Koyu et al. 2019^99^	PC- PG-RCT	Adults with metabolic syndrome (*n* = 40, mean age 52.5 yrs)	Kefir (~180 mL/day; traditional milk-grain fermentation)	Unfermented milk	12 weeks	Cardiometabolic biomarkers, gut microbiome	SCFA levels, inflammatory markers (TNF-α, IL-6)	Gut microbiome composition, SCFA changes	No significant between-group differences versus unfermented milk in gut microbiome composition or metabolic outcomes after 12 weeks. Within the kefir group, fasting insulin, HOMA-IR, TNF-α, IFN-γ and blood pressure improved, and Actinobacteria abundance increased, but these changes were not significantly different from the control group.	Microbiome profiling was included, but no significant between-group effects were observed, providing limited evidence for clinically meaningful gut-muscle benefits.
Zolghadrpour et al. 2024^93^	PC- PG-RCT	Adults with metabolic syndrome (*n* = 41, mean age 44.7 yrs)	Synbiotic yoghurt (containing strains of *Lactiplantibacillus plantaru*m, *Lactiplantibacillus pentosus* (2 × 10^8^ CFU), *Chloromyces marcosianos* yeast, and 3% of various natural plants (celery, shallot, chicory, and mint))	Control yoghurt without synbiotic components of intervention product	8 weeks	Metabolic syndrome components	Insulin sensitivity, inflammatory markers	Not assessed	Compared with regular yoghurt, synbiotic yoghurt significantly reduced fasting blood glucose (*p* = 0.005), fasting insulin (*p* = 0.001), HOMA-IR (*p* < 0.001), waist-to-hip ratio (*p* = 0.02) and systolic blood pressure (*p* = 0.008) after 12 weeks in adults with metabolic syndrome.	Metabolic benefits cannot be attributed specifically to fermentation because the intervention combined fermented dairy with probiotic and plant-derived components.
Chen et al. 2019^90^	PG-RCT	Obese women with NAFLD + metabolic syndrome (*n* = 92, mean age 48.9 yrs)	Conventional yoghurt (220 g/day; LAB matrix)	Standard milk	24 weeks	Metabolic and inflammatory markers	Insulin sensitivity; TNF-α; CRP; gut permeability markers	Gut microbiome composition assessed	Compared with milk, yoghurt reduced HOMA-IR (−0.53, *p* = 0.04), fasting insulin (−2.77 mU/L, *p* = 0.01), 2-h insulin (*p* < 0.001), intrahepatic lipid (−3.44%, *p* = 0.02), hepatic fat fraction (−3.48%, *p* = 0.02), ALT (−4.65 U/L, *p* = 0.02), serum LPS (−0.31 EU/mL, *p* < 0.001), TNF-α (*p* = 0.04), FGF21 (*p* < 0.001), and altered gut microbiome composition.	Suggests favourable metabolic, inflammatory and microbiome effects, but no direct skeletal muscle or protein synthesis outcomes were assessed.
Pei et al. 2017^100^	PG-RCT	Overweight/premenopausal women (*n* = 120, mean age 29.7 yrs)	Low-fat yoghurt, 339 g/day	Soy pudding (non-dairy control, energy- and nutrient-matched, non-fermented)	9 weeks	Inflammatory and metabolic markers	Insulin sensitivity; inflammatory cytokines	Not assessed	Yoghurt reduced TNF-α (*P* = 0.022) and TNF-α/sTNF-RII ratio (*P* = 0.001), increased IgM EndoCAb (*P* = 0.005), lowered the LBP:sCD14 ratio (*P* = 0.048), and increased PBMC expression of NFKBIA and TGFB1 in obese participants. No effects on IL-6, hsCRP or circulating LPS.	Provides indirect evidence for reduced inflammation and endotoxin exposure, but no muscle outcomes or microbiome profiling were performed.
Pražnikar et al. 2020^101^	CO-RCT	Overweight adults (*n* = 27, mean age 45.8 yrs)	Traditional kefir containing predominantly *Lentilactobacillus parakefiri*, *Lentilactobacillus kefiri*, *Lentilactobacillus kefiranofaciens* ssp. *kefirgranum*, with associated yeast species.	Standard milk	3 weeks	Gut permeability markers	Indirect metabolic relevance	Not assessed	ANCOVA-adjusted reduction in serum zonulin following kefir supplementation (*P* = 0.018), suggesting improved intestinal barrier function; glucose and lipid markers improved similarly following both kefir and milk.	Potentially relevant to muscle through improved barrier integrity, but no muscle-specific or broader anabolic and inflammatory outcomes were assessed.
Zheng et al. 2015^102^	CO-RCT	Healthy male adults (*n* = 15, ages 18–50 yrs)	Diet high in semihard cow’s cheese with equal amounts of dairy calcium (1.7 g/day)	Diet high in semi-skimmed milk (1.5% fat) or a butter-based control diet (no other dairy products)	14 days	SCFA production	Indirect metabolic relevance	Faecal SCFAs measured	Compared with milk, cheese consumption significantly increased faecal microbial metabolites including butyrate, propionate and malonate, increased urinary hippurate, and reduced urinary trimethylamine N-oxide (TMAO), choline, citrate, creatine and creatinine. Both dairy interventions increased faecal SCFA concentrations and faecal lipid excretion relative to the control diet, although cheese produced a distinct metabolomic profile from milk.	Demonstrates distinct microbial metabolite effects of fermented versus non-fermented dairy, but clinical, microbiome and skeletal muscle outcomes were not assessed.

*ALT* alanine aminotransferase, *ANCOVA* analysis of covariance, *CFU* colony-forming units, *CO-RCT* crossover randomised controlled trial, *CRP* C-reactive protein, *DB* double-blind, *EndoCAb* endotoxin-core antibody, *EU* endotoxin units, *FGF21* fibroblast growth factor 21, *FPF* fermented protein food, *GLP-1* glucagon-like peptide-1, *GMA* gut-muscle axis, *HbA1c* glycated haemoglobin, *HDL-C* high-density lipoprotein cholesterol, *HOMA-IR* homeostatic model assessment for insulin resistance, *hsCRP* high-sensitivity C-reactive protein, *IFN-γ* interferon-gamma, *IGF-1* insulin-like growth factor-1, *IgM* immunoglobulin M, *IL-6* interleukin-6, *LAB* lactic acid bacteria, *LBP* lipopolysaccharide-binding protein, *LDL-C* low-density lipoprotein cholesterol, *LPS* lipopolysaccharide, *MPS* muscle protein synthesis, *MPB* muscle protein breakdown, *NAFLD* non-alcoholic fatty liver disease, *NFKBIA* NF-kappa-B inhibitor alpha, *PBMC* peripheral blood mononuclear cell, *PC* placebo-controlled, *PG-RCT* parallel-group randomised controlled trial, *SCFA* short-chain fatty acid, *sCD14* soluble cluster of differentiation 14, *sTNF-RII* soluble tumour necrosis factor receptor II, *TGFB1* transforming growth factor beta 1, *TNF-α* tumour necrosis factor-alpha.

### Direct skeletal muscle-related outcomes

To date, a small number of published RCTs have directly evaluated skeletal muscle-related outcomes following interventions involving FPFs (Table 1), and each differs substantially in intervention design and mechanistic scope.

Kim et al.^97^ randomised 48 healthy middle-aged Korean men (mean age 44.8 years) to consume fermented whey protein produced using *Lacticaseibacillus casei* DK211 or an isonitrogenous non-fermented whey protein twice daily for eight weeks alongside a structured exercise programme. Between-group analysis demonstrated significantly greater improvements in dynamic balance in the fermented whey group (both left and right limbs; two-way ANOVA, *p* = 0.032), whereas changes in grip strength, lean mass, back strength and other physical performance outcomes did not differ significantly between interventions despite several within-group improvements observed following fermented whey supplementation. Although these findings suggest that fermentation may enhance selected aspects of neuromuscular performance, the overall magnitude of benefit was modest and evidence for superior anabolic effects over conventional whey protein remains limited. Another aspect to consider is that participants were Korean, middle-aged men and therefore limits generalisability to older adults, women and other ethnic groups. Furthermore, all participants completed a structured exercise programme, making it difficult to isolate the independent contribution of fermentation. Neither gut microbiome composition, microbial metabolites nor metrics of muscle anabolism were assessed, also precluding mechanistic interpretation.

Jung et al.^98^ evaluated a kefir-derived postbiotic beverage generated using *Lentilactobacillus kefiri* DH5 fermentation products combined with whey protein in 53 healthy adults (mean age 54.6 years) consuming 6 g/day for 12 weeks. Participants receiving the intervention demonstrated a significant within-group improvement in dominant-hand grip strength (intention-to-treat (ITT) *p* = 0.012; per-protocol (PP) *p* = 0.025), whereas the placebo group showed no significant within-group change. However, the between-group difference in grip strength was not statistically significant (ITT *p* = 0.498; PP *p* = 0.363), and no significant differences were observed for lean mass, bone mineral measures or circulating IGF-1. The intervention was accompanied by alterations in gut microbial composition, including increased relative abundance of *Faecalibacterium* and *Latilactobacillus*, together with reductions in circulating IL-1β and increases in plasma irisin within the intervention group. Although these findings provide preliminary mechanistic support for interactions between fermentation-derived postbiotics, the gut microbiome and muscle-related physiology, the absence of significant between-group differences for the primary functional outcome means that evidence for a clinically meaningful effect on muscle performance remains tentative. Furthermore, because the intervention consisted primarily of fermentation-derived postbiotics rather than an intact FPF matrix, extrapolation to habitual dietary FPF consumption should be made cautiously.

Exercise recovery has also been investigated following ingestion of *Lactobacillus helveticus*-fermented milk. In a double-blind crossover RCT involving 18 healthy young men (mean age 21.6 years), participants consumed 600 mL/day of fermented milk (providing approximately 6.6 g protein) or an isonitrogenous non-fermented milk control before and after a bout of high-intensity resistance exercise^91^. Compared with the control beverage, fermented milk significantly reduced post-exercise muscle soreness (total soreness score: 12.6 ± 1.1 vs. 14.2 ± 1.2; *p* < 0.05) and attenuated the exercise-induced decline in respiratory quotient and carbohydrate oxidation, suggesting improved post-exercise glucose utilisation. Serum creatine phosphokinase concentrations also tended to be lower following fermented milk consumption, although this difference did not reach statistical significance. However, the study assessed only acute recovery responses following a single exercise bout, and neither MPS, gut microbiome composition nor mechanistic biomarkers were evaluated. Consequently, while these findings support a potential role for fermented milk in exercise recovery, they provide only indirect evidence regarding longer-term skeletal muscle adaptations or GMA mechanisms.

Collectively, the available evidence suggests that selected FPF interventions may confer modest improvements in certain functional measures of muscle performance, particularly grip strength, balance and exercise recovery. However, these findings should be interpreted cautiously. The current evidence base comprises a small number of heterogeneous RCTs involving relatively small samples of predominantly healthy adults, and statistically significant between-group improvements in direct muscle outcomes have been inconsistent across studies. Furthermore, several interventions incorporated relevant co-interventions, including structured exercise programmes or postbiotic formulations rather than whole FPFs, making it difficult to isolate the independent effects of FPFs. Importantly, no published RCT comprising fermented versus non-fermented protein consumption has directly assessed MPS nor upstream signalling mechanisms by way of methodologies such as stable isotope tracer methodologies and biopsy-derived anabolic signalling (e.g., mTORC1 activation), as well as high-resolution imaging-based changes in muscle morphology. Consequently, current evidence supports biological plausibility but remains insufficient to establish that FPFs directly enhance skeletal muscle anabolism in humans.

### Indirect muscle and immunological outcomes

A larger body of RCT evidence demonstrates that FPFs influence metabolic and inflammatory pathways related to skeletal muscle physiology, even when muscle-specific outcomes were not assessed directly (Table 2).

Several RCTs have reported improvements in glycaemic regulation and insulin sensitivity following fermented dairy interventions. In adults with type 2 diabetes, daily consumption of 600 mL probiotic kefir for eight weeks significantly reduced HbA1c compared with conventionally fermented milk and produced a greater reduction in fasting glucose after adjustment for baseline values^92^. Similarly, in adults with metabolic syndrome, daily intake of 300 g synbiotic yoghurt containing *Lactiplantibacillus plantarum*, *Lactiplantibacillus pentosus* and *Chloromyces marcosianos* yeast for 12 weeks significantly reduced fasting glucose, fasting insulin and HOMA-IR compared with regular yoghurt, alongside modest improvements in systolic blood pressure and waist-to-hip ratio^93^. Conventional fermented dairy products have also demonstrated metabolic benefits. In obese women with non-alcoholic fatty liver disease and metabolic syndrome, 220 g/day yoghurt for 24 weeks reduced fasting insulin, HOMA-IR, postprandial insulin responses and hepatic fat compared with milk^90^. By contrast, a smaller 12-week kefir trial in adults with metabolic syndrome reported within-group improvements in fasting insulin and HOMA-IR, but these did not differ significantly from the unfermented milk control^99^. Collectively, these studies suggest that fermented dairy products may improve metabolic regulation and insulin sensitivity, although the magnitude of benefit appears to vary across populations and interventions.

Several studies also report favourable effects on inflammatory and immune-related biomarkers. In adults with metabolic syndrome, kefir consumption reduced circulating TNF-α and IFN-γ concentrations, although these changes were not significantly greater than those observed with unfermented milk^99^. In obese women with NAFLD and metabolic syndrome, yoghurt significantly reduced circulating TNF-α, LPS, fibroblast growth factor 21 and triglycerides while increasing antioxidant enzyme activity compared with milk^90^. Similarly, nine weeks of low-fat yoghurt consumption reduced TNF-α and the TNF-α:sTNF-RII ratio, increased circulating endotoxin-core antibodies, lowered the LBP:sCD14 ratio and upregulated expression of anti-inflammatory genes including NFKBIA and TGFB1 compared with a non-fermented soy control in premenopausal women^100^. Given the established inhibitory effects of chronic low-grade inflammation on insulin signalling, PI3K/Akt activation and mTOR-mediated protein synthesis, these findings provide biological plausibility for indirect benefits to skeletal muscle via inflammatory and immune pathways.

Gut barrier integrity has received comparatively limited investigation. In overweight adults, three weeks of kefir supplementation reduced circulating zonulin, a marker of intestinal permeability, after adjustment for baseline values, whereas milk produced no comparable effect^101^. In parallel, yoghurt consumption has been shown to reduce circulating LPS and improve markers of endotoxin handling, including the LBP:sCD14 ratio, consistent with reduced metabolic endotoxemia^90,100^. Although these observations align with proposed mechanisms linking the GMA to muscle metabolism, no study has directly demonstrated that improvements in gut barrier function following FPF consumption translate into enhanced MPS, muscle mass or physical function.

Taken together, current human evidence suggests that FPFs can favourably influence systemic metabolic regulation, inflammatory status and aspects of gut barrier function. However, these studies primarily evaluate circulating biomarkers rather than skeletal muscle outcomes, and positive findings are not consistently observed across all trials. Consequently, whether these immunometabolic adaptations translate into meaningful improvements in muscle mass, strength or anabolic responsiveness remains to be established.

### Microbiome and mechanistic outcomes

Relatively few RCTs have comprehensively characterised gut microbial responses to FPF consumption, with an absence of investigations simultaneously assessing skeletal muscle outcomes.

Among the available evidence, several RCTs described above reported increases in SCFA-producing taxa or elevations in faecal SCFA concentrations following FPF consumption^90,98,99^. In addition, in an RCT of 15 healthy men (18–50 years), consumption of a high-cheese diet for 14 days significantly increased faecal butyrate, propionate and malonate, alongside higher urinary hippurate, compared with an isocaloric milk diet, suggesting enhanced microbial fermentation and altered host-microbial metabolism^102^ (Table 2). However, microbial findings remain highly heterogeneous owing to differences in sequencing methodologies, taxonomic resolution, analytical pipelines and reporting of functional microbial outputs.

An important element of causally evaluating modulation of the GMA as a facet to improve skeletal muscle health is that modulation of microbiome composition should not be interpreted as mechanistic evidence of the potential to induce improvements in skeletal muscle physiology. In this context, the PROMOTe RCT enrolled 72 community-dwelling older adults (36 twin pairs; mean age 73 years) who completed 12 weeks of daily prebiotic supplementation (inulin and fructo-oligosaccharides) or placebo, with both groups also receiving BCAA supplementation and prescribed resistance exercise^103^. Although the prebiotic significantly altered gut microbiome composition, including increased relative abundance of *Bifidobacterium*, and improved cognitive performance assessed via the Cambridge Neuropsychological Test Automated Battery (CANTAB) compared with placebo (*p* = 0.014), it produced no improvement in the primary muscle-related outcome of chair-rise time (*p* = 0.494) or other physical performance measures. Although this intervention did not involve FPFs directly, it highlights this important principle that microbiome modulation, even alongside resistance exercise and amino acid supplementation, may not be sufficient to augment skeletal muscle physiology and function. Rather, alterations in microbial metabolic activity, including production of SCFAs and other bioactive metabolites, are more likely to represent the biologically relevant mediators. However, few published studies have concurrently quantified microbial metabolites, host metabolomics and skeletal muscle outcomes, limiting mechanistic interpretation.

## Overall appraisal of clinical evidence

Several important limitations should be considered when interpreting the current literature. First, intervention heterogeneity is substantial, encompassing fermented whey protein, yoghurt, kefir, fermented milk, synbiotic products and postbiotic formulations, with protein doses ranging from approximately 6 g/day to >70 g/day, intervention durations varying from a single post-exercise feeding to 24 weeks, and microbial exposures differing markedly across studies. Although most studies report the amount of FF provided, viable microbial counts (CFU) remain inconsistently characterised, limiting assessment of dose-response relationships and translation to habitual dietary intake.

Second, many interventions incorporate co-interventions including structured resistance exercise. Such factors likely interact synergistically with FPFs, making it difficult to isolate the independent contribution of FPFs. Current evidence may therefore reflect enhancement of exercise-induced or nutrition-induced adaptations rather than direct anabolic effects attributable solely to FPF consumption.

Third, the clinical evidence comprises a mixture of positive, neutral and indirect findings. Improvements in grip strength, physical performance or metabolic biomarkers have been reported, yet other studies have demonstrated no statistically significant improvements in muscle-related outcomes. Such inconsistency hampers the ability to draw firm conclusions.

Fourth, most studies rely on proxy outcomes including grip strength, physical performance tests, inflammatory biomarkers, insulin sensitivity or microbiome composition. While clinically relevant, these measures cannot establish whether FPFs directly enhance MPS, upstream signalling events, or muscle remodelling. Notably, no human studies have yet combined FPF interventions with stable isotope tracers, skeletal muscle biopsy, and integrated microbiome-metabolome analyses capable of directly demonstrating GMA-mediated anabolic effects.

Collectively, the available clinical evidence supports biological plausibility that FPFs may influence skeletal muscle through multiple complementary pathways involving microbial metabolites, systemic inflammation, endocrine regulation and improved protein quality and anabolic substrate availability. However, direct evidence demonstrating that fermentation itself enhances MPS or produces clinically meaningful improvements in muscle mass and function independent of co-interventions remains limited. Accordingly, current evidence should be regarded as promising but preliminary, highlighting the need for well-controlled RCTs integrating microbiome characterisation with direct skeletal muscle phenotyping.

## Fermented protein foods versus probiotic supplementation

In comparison to FPFs, probiotics have received substantially greater attention concerning GMA modulation, although it is important to note that the two approaches should not be considered interchangeable. Probiotic supplementation typically delivers well-characterised microbial strains at defined doses, allowing precise control over microbial exposure and facilitating quantitative synthesis across RCTs. Indeed, several recent systematic reviews and meta-analyses of RCTs have reported statistically significant improvements in muscle strength, with some also demonstrating modest improvements in muscle mass following probiotic supplementation, particularly in older adults and clinical populations, although heterogeneity between strains and interventions remains^104–106^. Consequently, the current evidence base supporting probiotics is considerably more mature than that for FPFs.

By comparison and as indicated above, human intervention studies investigating FPFs remain relatively few, highly heterogeneous and have rarely been designed specifically to examine GMA mechanisms. Direct comparisons between the two approaches are therefore not currently possible. Nevertheless, FPFs differ conceptually from probiotic supplements because they provide considerably more than live microorganisms alone. Fermentation modifies the physicochemical properties of the protein matrix while simultaneously generating bioactive peptides, organic acids, and other microbial metabolites that may influence digestion, nutrient bioavailability, host metabolism and immune function. These matrix-dependent effects may act synergistically with microbial activity to influence skeletal muscle physiology through mechanisms extending beyond those achievable with isolated probiotic strains.

Important differences also exist regarding microbial delivery. Probiotic supplements generally provide standardised CFU doses of selected strains, whereas microbial composition within FPFs varies according to starter cultures, fermentation conditions, storage and food processing. Although this variability complicates standardisation and dose-response evaluation, it may also expose consumers to a broader range of microbial taxa and fermentation-derived metabolites. However, evidence demonstrating durable gut colonisation following FPF consumption remains limited, and probiotic supplements would appear to primarily exert effects through transient microbial exposure and metabolite production rather than permanent microbial engraftment^15,107,108^.

Regulatory frameworks also differ. Probiotic supplements are typically marketed as dietary supplements or functional foods with defined microbial compositions and quality control standards, whereas FPFs are generally consumed as conventional foods with long histories of safe dietary use but substantially less standardisation of microbial content. These differences have important implications for reproducibility across clinical studies and translation into dietary recommendations.

From a translational perspective, FPFs may therefore represent a complementary rather than competing strategy to probiotic supplementation. Unlike probiotic capsules, FPFs simultaneously deliver high-quality dietary protein together with fermentation-derived bioactive compounds within an established food matrix. This dual nutritional and microbial functionality may offer practical advantages for populations already requiring increased protein intake, including older adults, athletes and individuals recovering from illness and/or injury. However, whereas probiotic interventions benefit from a substantially larger body of quantitative clinical evidence, comparable evidence supporting FPFs remains preliminary and insufficient to determine whether fermentation confers benefits beyond those attributable to high-quality protein or probiotic supplementation alone. Accordingly, FPFs should presently be regarded as a promising but less well-characterised nutritional strategy rather than an alternative to probiotic supplementation. Future adequately powered trials directly comparing FPFs with strain-specific probiotic interventions, while incorporating standardised microbial characterisation, quantitative metabolomics and direct skeletal muscle outcomes, will be required to determine whether fermentation of the food matrix confers benefits beyond those attributable to probiotic administration or high-quality protein intake alone.

## Translational insights and practical implications

Taking the current landscape of FPF RCT evidence into consideration highlights several priorities for advancing the field (Box 1).

Despite the current limitations in direct clinical evidence, the mechanistic and emerging human data suggest that FPFs may hold translational potential for diverse populations at risk of muscle loss or in need of optimised recovery. Older adults, who often experience anabolic resistance and sarcopenia, may also exhibit reduced dietary diversity due to physiological, sensory, and appetite-related changes, which may in turn contribute to reduced gut microbiome diversity and functional capacity^3,63^. Accordingly, they may represent a priority population in which these mechanisms warrant further investigation, although this remains to be confirmed in adequately powered RCTs. Athletes may also represent an important population in which these mechanisms warrant further investigation, particularly with respect to recovery, performance, and attenuation of exercise-induced inflammatory stress^8^. Similarly, individuals with metabolic or gastrointestinal disorders, who frequently exhibit altered microbiome composition and impaired nutrient absorption, may also benefit from targeted FPF interventions^67^. Another population in which FPFs may offer a therapeutic option in recovery is individuals who have suffered a spinal cord injury, in which muscle atrophy is a significant consequence^109^, alongside perturbation of gut microbial ecology and function^110^, thereby introducing a model in which skeletal muscle maintenance is paramount in recovery, with a backdrop of GMA disruption.

Food characteristics and fermentation conditions may also influence functional outcomes. Dairy-based matrices such as yoghurt, kefir, and cheese differ in microbial composition, metabolite profiles, and protein structure, while emerging plant-based fermented alternatives offer distinct bioactive potentials. Recognising the matrix as a functional delivery system, rather than solely a source of protein, highlights the importance of tailoring fermentation strategies to optimise the release of bioactive peptides, SCFAs, and other metabolites relevant to muscle metabolism.

Collectively, the available mechanistic evidence suggests that fermentation has the potential to enhance skeletal muscle health through multiple complementary pathways, including improving protein bioavailability and amino acid kinetics, modulating host-microbe crosstalk to favour anti-inflammatory and anabolic signalling, and shaping the systemic metabolic environment to support MPS. While these mechanisms provide a biologically plausible rationale for FPF-based interventions, direct clinical evidence demonstrating meaningful improvements in muscle mass, strength or anabolic responsiveness remains limited. Future work should therefore focus on determining which fermented protein matrices, microbial compositions and target populations are most likely to derive clinically relevant benefit, thereby supporting the development of next-generation FPFs as precision nutrition strategies that bridge food science, microbiome modulation and skeletal muscle physiology.

Box 1 Suggested future directions to advance fermented protein foods gut-muscle axis research

- Conduct stable isotope tracer methodologies to compare the effects on MPS of fermented vs non-fermented protein matrices.
- Incorporate endpoints relevant to cellular signalling (e.g. mTORC activation) in muscle samples to elucidate the effects on upstream anabolic/catabolic regulators.
- Incorporate endpoints relevant to skeletal muscle morphology and architecture using methodologies such as Dual-Energy X-ray Absorptiometry (DXA) and Magnetic Resonance Imaging (MRI).
- Standardise and thoroughly characterise microbial composition across fermented protein interventions.
- Integrate multi-omics approaches to associate microbial metabolites with muscle molecular signalling.
- Perform microbiome-stratified analyses to determine individual variability in anabolic response.
- Establish dose-response relationship to optimise fermented protein intake for skeletal muscle health outcomes.
- Conduct tissue-specific investigations comparing skeletal muscle responses with those of cardiac muscle, adipose tissue and liver to determine the selectivity of fermented protein-mediated gut-host signalling.
- Implement RCTs in at-risk groups with regard to both skeletal muscle and gastrointestinal physiology (e.g. sarcopenic older adults exhibiting anabolic resistance and/or gut dysbiosis, sufferers of spinal cord injury exhibiting significant muscle atrophy and GMA disruption) to determine the impact in physiologically constrained models.

## Future directions: a conceptual framework for precision fermented protein nutrition

Despite growing mechanistic evidence, clinical evidence directly supporting FPF-induced skeletal muscle adaptations remains limited. Few RCTs have been designed specifically to evaluate skeletal muscle outcomes, and there is an absence of gold-standard phenotyping techniques such as stable isotope tracer methodologies in MPS measurement, elucidation of upstream regulators via muscle biopsy characterisation, or high-resolution imaging. Consequently, establishing causal links between FPF intake, microbiome modulation and consequences in skeletal muscle such as augmentation of MPS and muscle mass remains an important research question.

Future studies should seek to reduce the considerable heterogeneity that currently characterises FPF interventions, including differences in food matrices, microbial composition, fermentation conditions, viable microbial content and protein characteristics. Greater standardisation of intervention reporting would facilitate quantitative comparison across studies and help identify which components of FPFs are most relevant to skeletal muscle health.

Similarly, integration of multi-omics approaches, including metagenomics, metabolomics, proteomics and host molecular phenotyping, offers considerable potential to elucidate the biological pathways connecting fermentation-induced microbial activity to skeletal muscle physiology.

Taking the mechanistic plausibility and current state of human evidence, we propose a conceptual framework exploring the potential of precision fermented protein nutrition to guide future investigation. This framework recognises FPFs as complex biological systems comprising modified protein matrices, live microorganisms and/or components thereof, and fermentation-derived bioactive compounds that may collectively influence host skeletal muscle physiology. It is intended to provide a framework for hypothesis generation and study design. Within this framework, future mechanistic studies could investigate whether specific fermented protein matrices can be tailored to specific populations, such as older adults, athletes, individuals with metabolic dysfunction or sufferers of conditions and/or injuries associated with muscle atrophy, while incorporating comprehensive microbiome characterisation together with direct skeletal muscle phenotyping. Such studies will be necessary to determine whether fermentation of protein foods provides benefits beyond those attributable to protein or probiotic supplementation alone.

## Conclusions

FPFs represent a biologically plausible but currently underexplored strategy for modulating skeletal muscle health through GMA mechanisms (Fig. 3). Mechanistically, they combine bioactive peptides, live microbes and/or components thereof, as well as fermentation-derived bioactive compounds that have the potential to enhance protein digestibility and modulate multiple pathways coordinating inflammatory, immune, endocrine, metabolic and anabolic processes which converge to influence host skeletal muscle physiology. Although emerging human studies support favourable effects on metabolic health, inflammatory and intestinal barrier regulation, microbial composition and limited markers of physical function, direct evidence demonstrating improvements in MPS, muscle mass or muscle function is absent.

This review highlights several important gaps in the current literature, including the scarcity of adequately powered RCTs incorporating rigorous skeletal muscle phenotyping, inconsistent characterisation of FPF interventions, and the limited integration of microbiome, metabolomic and host molecular analyses. Addressing these limitations will be essential before causal relationships between FPF consumption and skeletal muscle health can be established. We propose that future research should consider FPFs as complex nutritional matrices rather than merely sources of dietary protein or probiotics. Integrating food matrix biology, microbial ecology and skeletal muscle physiology may provide new mechanistic insights into host-microbe interactions relevant to muscle health.

Ultimately, determining whether FPFs confer clinically meaningful advantages over conventional protein sources or pro-, pre-, syn- and postbiotic supplementation will require adequately powered, mechanistically informed RCTs capable of distinguishing the independent contributions of the FPF matrix, microbial components and protein substrate. Such investigations will determine whether FPFs have a future role in precision nutrition strategies aimed at ameliorating skeletal muscle health across the lifespan.
